# Insulator speckles associated with long-distance chromatin contacts

**DOI:** 10.1242/bio.019455

**Published:** 2016-07-27

**Authors:** Melanie K. Buxa, Johan A. Slotman, Martin E. van Royen, Maarten W. Paul, Adriaan B. Houtsmuller, Rainer Renkawitz

**Affiliations:** 1 Institute for Genetics, Justus-Liebig-University, Heinrich-Buff-Ring 58, Giessen D35392, Germany; 2 Department of Pathology, Josephine Nefkens Institute, Erasmus Optical Imaging Centre, Erasmus MC, Postbus 2040, Rotterdam 3000 CA, The Netherlands

**Keywords:** dCTCF, CP190, Insulator, Speckle, Polycomb bodies

## Abstract

Nuclear foci of chromatin binding factors are, in many cases, discussed as sites of long-range chromatin interaction in the three-dimensional nuclear space. Insulator binding proteins have been shown to aggregate into insulator bodies, which are large structures not involved in insulation; however, the more diffusely distributed insulator speckles have not been analysed in this respect. Furthermore, insulator binding proteins have been shown to drive binding sites for Polycomb group proteins into Polycomb bodies. Here we find that insulator speckles, marked by the insulator binding protein dCTCF, and Polycomb bodies show differential association with the insulator protein CP190. They differ in number and three-dimensional location with only 26% of the Polycomb bodies overlapping with CP190. By using fluorescence *in situ* hybridization (FISH) probes to identify long-range interaction (kissing) of the *Hox* gene clusters Antennapedia complex (ANT-C) and Bithorax complex (BX-C), we found the frequency of interaction to be very low. However, these rare kissing events were associated with insulator speckles at a significantly shorter distance and an increased speckle number. This suggests that insulator speckles are associated with long-distance interaction.

## Abstract (precis)

**Summary:** Insulator speckles have been discussed to be either storage places for insulator proteins or to represent sites of long-range chromatin interaction. Here we demonstrate their association with interacting chromatin.

## INTRODUCTION

Long-range chromatin contacts occur in three-dimensional structures, which enable interactions of remotely located chromosomal regions. These specific interactions are mediated by biophysical and biochemical features such as protein-protein interactions (Fullwood et al., 2009). The insulator-binding protein CTCF has been found to demarcate chromatin domains and to influence proper gene expression by interfering with the cross-talk between promoters and regulatory elements (for review see Ali et al., 2016). A further feature of long-range contacts is the involvement of transcriptionally active genes. Co-regulated genes preferentially cluster at transcription factories that seem to be optimized for their high-level transcription (Noordermeer et al., 2011; Schoenfelder et al., 2010).

One well-studied long-range interaction between co-regulated genes in *Drosophila melanogaster* has been analysed by fluorescence *in situ* hybridization (FISH) described as ‘*Hox* gene kissing’ (Bantignies et al., 2011). In flies, the two *Hox* gene clusters, the Antennapedia complex (ANT-C) and the Bithorax complex (BX-C), are located on the same chromosome (3R) and are separated by approximately 10 Mb of euchromatic sequences. Bantignies et al. (2011) provide evidence that the two distant *Hox* complexes can interact within nuclear Polycomb group (PcG) bodies in tissues where they are co-repressed; moreover, this colocalization increases during development and depends on PcG proteins. By using three-dimensional (3D) DNA FISH, they could detect this *Hox* gene kissing in diploid interphase nuclei at a significant frequency. Furthermore, chromosome conformation capture (3C), circularized chromosome conformation capture (4C) and the 3C derivate Hi-C validated this observation (Bantignies et al., 2011; Lanzuolo et al., 2007; Sexton et al., 2012). *Drosophila* PcG bodies are microscopically visible nuclear foci in which Polycomb target genes co-localize suggesting an organization in 3D.

Divergent from the above studies, (Li et al., 2011) have shown that insulators, not Polycomb response elements (PREs), are required for long-range interactions between Polycomb targets and thereby Polycomb bodies may be formed. PcG-regulated genes are targeted by insulator proteins to different nuclear structures depending on their state of activity (Li et al., 2013). In Drosophila, 9 insulator-binding proteins (IBPs) have been identified (Ali et al., 2016), including Su(Hw) (Parkhurst et al., 1988), GAG-binding factor GAF (Ohtsuki and Levine, 1998), Zw5 (Gaszner et al., 1999) and BEAF-32 (Roy et al., 2007; Zhao et al., 1995). In addition to these DNA binding factors, Mod(mdg4)67.2 (Buchner et al., 2000) and Centrosomal protein 190 (CP190) (Pai et al., 2004) are physically and functionally connected to insulators without binding directly to DNA.

*Drosophila* insulator proteins co-localize in discrete foci, named insulator bodies, in the interphase cell nucleus. Previous publications suggested that *Drosophila* insulator bodies are large nuclear structures with 5 to 25 insulator bodies per nucleus, marked by the insulator factors Su(Hw), Mod(mdg4)67.2 (Gerasimova et al., 2000; Gerasimova and Corces, 1998), CP190 (Pai et al., 2004) and dCTCF (Gerasimova et al., 2007). The results suggested that insulator factors work together to form insulator bodies and act as contact sites of functional insulators in 3D within the *Drosophila* interphase nucleus. Contrasting reports show that insulator bodies do not function as connecting sites of insulators, rather they are aggregated proteins not involved in insulation (Golovnin et al., 2008, 2012). Furthermore, it has been shown that insulator bodies form in response to osmotic stress through the coalescence of diffusely distributed speckles (Schoborg et al., 2013). In contrast to the large insulator bodies, the much refined and delicate appearance of insulator speckles is not yet well analysed. They do co-localize with Su(Hw), CP190, Mod(mdg4), BEAF and EAST (Golovnin et al., 2015; Schoborg et al., 2013), but it has not been shown whether they are sites of long-distance chromatin interaction.

Here we wanted to test whether the refined insulator speckles are associated with sites of long-distance chromatin interaction. As model sites we used the *Drosophila Hox* genes for which interaction has been demonstrated as kissing events and as contacting regions in chromosome conformation capture assays (Bantignies et al., 2011; Sexton et al., 2012). We used FISH combined with immunostaining (FISH-I) and found that *Hox* gene kissing is a rare event, but that dCTCF insulator speckles are located significantly closer to contacting *Hox* genes in comparison to non-contacting cases.

## RESULTS

### *Hox* gene kissing is a rare event in wing and eye imaginal discs

**Fig. 1.**
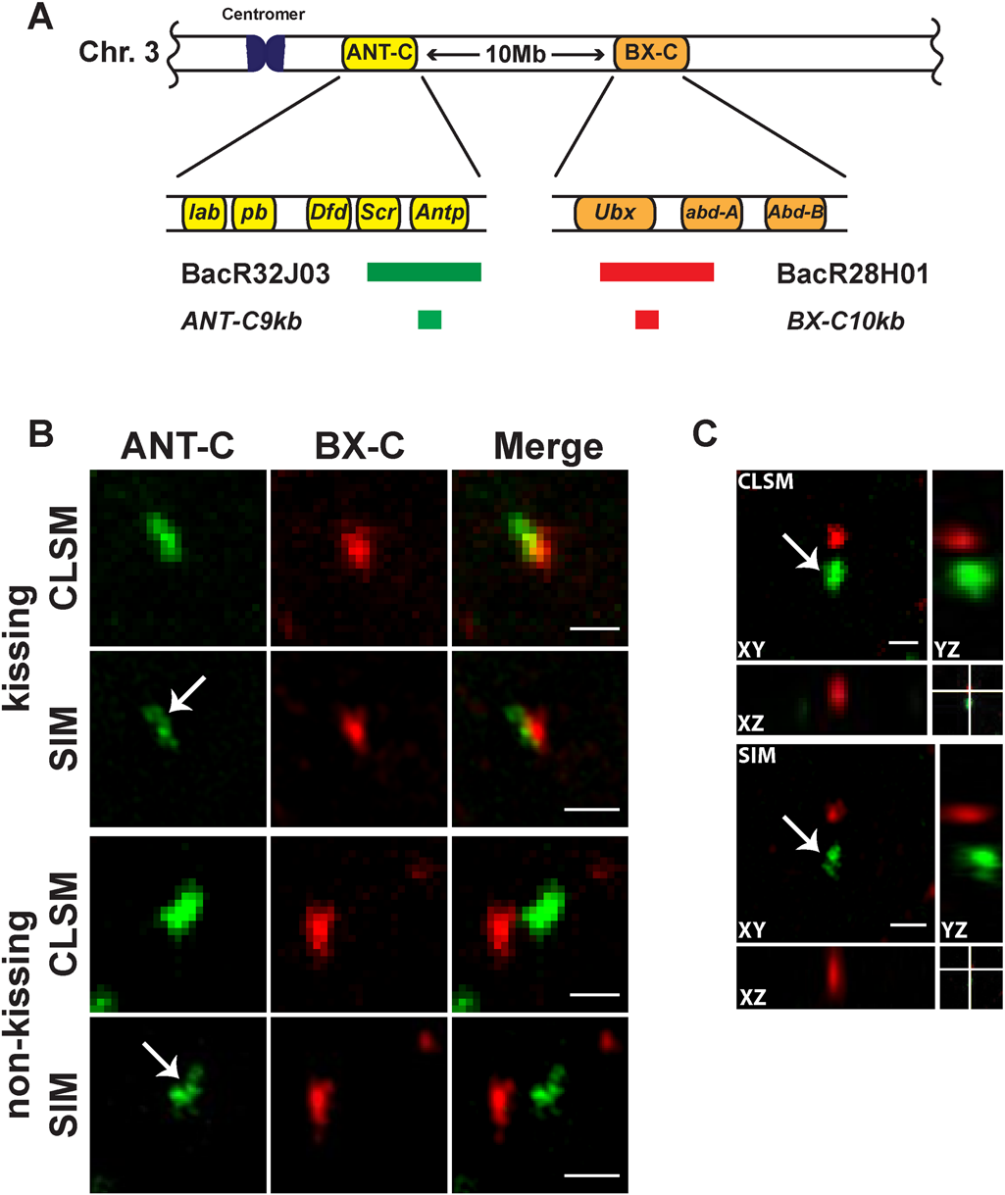
**Long DNA FISH probes result in non-contiguous staining detected by structured illumination microscopy (SIM).** (A) Schematic illustration of the Antennapedia complex (ANT-C) and the Bithorax complex (BX-C) on the right arm of chromosome 3 (3R) in *Drosophila melanogaster*. The DNA FISH probes are highlighted either in green, representing parts of ANT-C, or in red, representing parts of BX-C. For the exact genomic locations of each probe see Table S1. (B) DNA FISH with long probes in wing imaginal discs imaged either with confocal laser scanning microscopy (CLSM) or with structured illumination microscopy (SIM). Increased resolution with SIM revealed the structured and incomplete appearance of the FISH probes in the case of kissing and non-kissing *Hox* genes (white arrows). Kissing was defined as distances between probe pair centres shorter than 350 nm (Bantignies et al., 2011). The scale bars represent 1 µm. (C) Orthogonal view of DNA FISH in wing imaginal discs displaying increased lateral (XY) and axial (XZ and YZ) resolution with SIM as compared to CLSM. Scale bars: 1 µm.

**Fig. 2.**
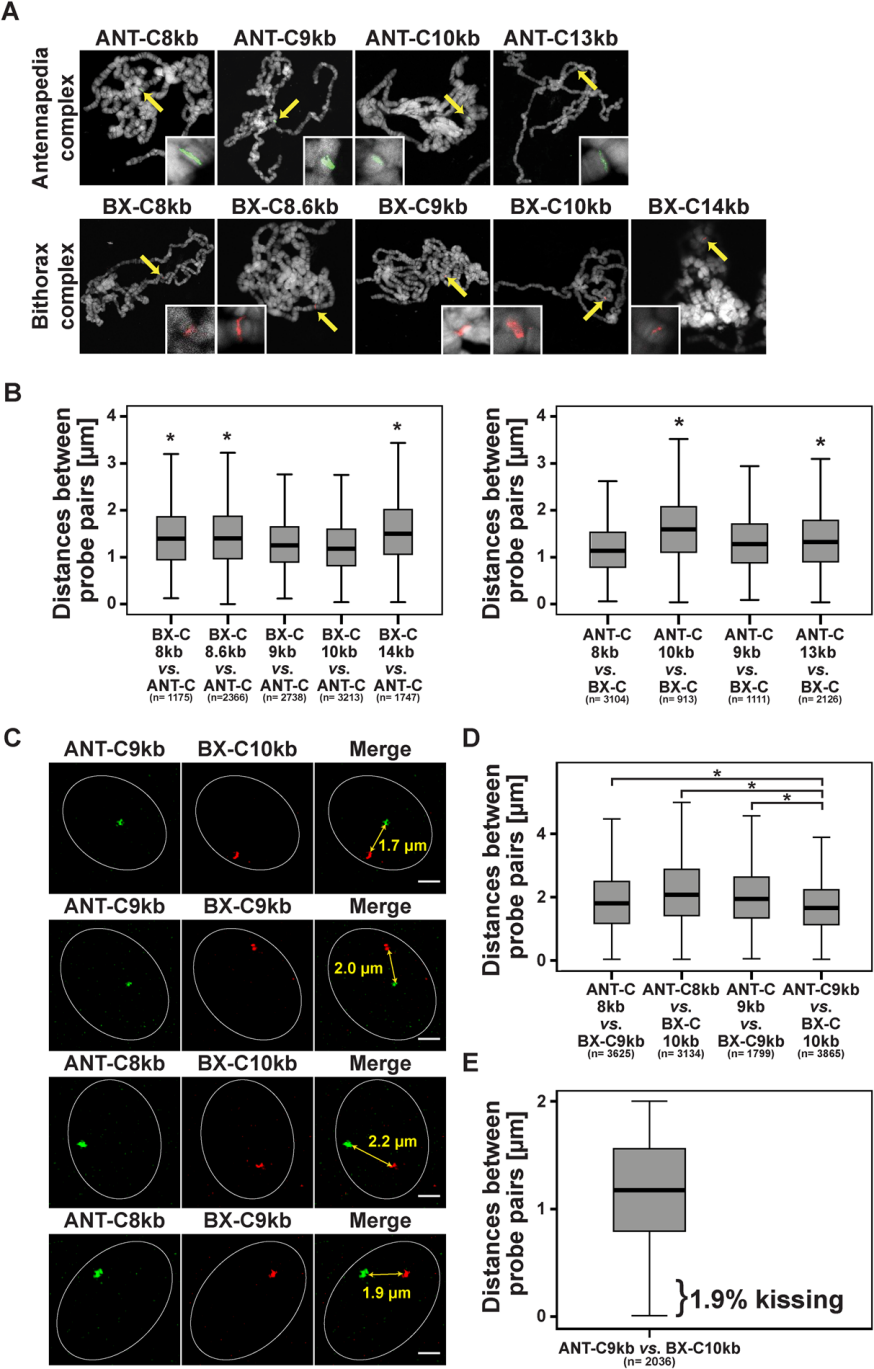
**Probe pair ANT-C9kb/BX-C10kb is in closest proximity to each other.** (A) Specificity of short probes verified by DNA FISH on polytene chromosomes. Each of the probes showed a distinct single band as magnified in the inset. (B) Hybridization of each of the short probes of one *Hox* gene cluster together with the long probe of the other *Hox* gene cluster revealed small but significant differences in distance. BX-C9kb and BX-C10kb as well as ANT-C8kb and ANT-C9kb showed the shortest distances to their corresponding long probe. Significant differences (two-tailed Mann–Whitney-U test) in distance are indicated (*). (C) Hybridization of one of the ANT-C probes ANT-C8kb or ANT-C9kb with one of the BX-C probes BX-C9kb or BX-C10kb. Examples of nuclei representing the mean distances between the short probes. Mean distances are highlighted in the merge view. Scale bars: 1 µm. Imaged with SIM. (D) Box-plot mean values indicated that the probe pair ANT-C9kb/BX-C10kb showed a significantly (two-tailed Mann-Whitney-U test) closer distance than the others, indicated (*). (E) Distance between ANT-C.9kb and BX-C.10kb in wing imaginal disc nuclei. *Hox* gene kissing occurred in only 1.9% of the nuclei. *n*=total number of analysed wing imaginal disc nuclei in B, D and E. For *P* values of the pairwise comparison in B and D see Table S2.

In order to analyse *Hox* gene kissing in more detail and in high resolution, we performed DNA FISH and used structured illumination microscopy (SIM) in comparison to confocal laser scanning microscopy (CLSM). *Hox* gene kissing has been described as the visual association of the *Antennapedia* (*Antp*) gene from the Antennapedia complex ANT-C and the *Ultrabithorax* (*Ubx*) gene from the Bithorax complex BX-C (Bantignies et al., 2011). The two complexes are located on the right arm of chromosome 3, separated by 10 Mb of DNA (Fig. 1A). We used labelled BAC probes, which contain either parts of ANT-C (BacR32J03) or parts of the BX-C (BacR28H01). The BAC probes cover 190 kb and 140 kb of the two complexes, respectively. The increased resolution achieved through SIM leads to the detection of long and discontinuous signals (Fig. 1B,C; arrows). In view of the fact that in *Drosophila melanogaster* homologues chromosomes are essentially paired in all somatic cells throughout development (Metz, 1916), one should expect a single labelled spot for each probe. We assume that the high SIM resolution allows detecting irregularities in probe labelling and/or hybridization. Therefore, we reduced the size of the probes to about 10 kb. The specificity of the short probes was tested by FISH on polytene chromosomes resulting in single bands for each of the short probes (Fig. 2A).

Given the fact that the close vicinity of the two *Hox* gene clusters in the three-dimensional space results in the observation of *Hox* gene kissing (Bantignies et al., 2011), we wanted to identify those sequences and probes which result in the closest distance in the interphase nuclei. Therefore, we determined which of the short probes shows the shortest distances to the long probe of the other *Hox* gene cluster (Fig. 2B). Despite the fact that the differences are not dramatic, two probes from BX-C and two from ANT-C showed significant shorter distances from the respective long probe. BX-C9kb and BX-C10kb as well as ANT-C8kb and ANT-C9kb are closer to the respective long probe. Next we hybridized all combinations of these four short probes. Of these, ANT-C9kb and BX-C10kb are in closest proximity to each other (Fig. 2D). The mean distance is 1.7 µm for the ANT-C9kb/BX-C10kb combination, which is significantly shorter than the other combinations (Fig. 2D). Therefore, for the following analyses we used this probe pair combination.

**Fig. 3.**
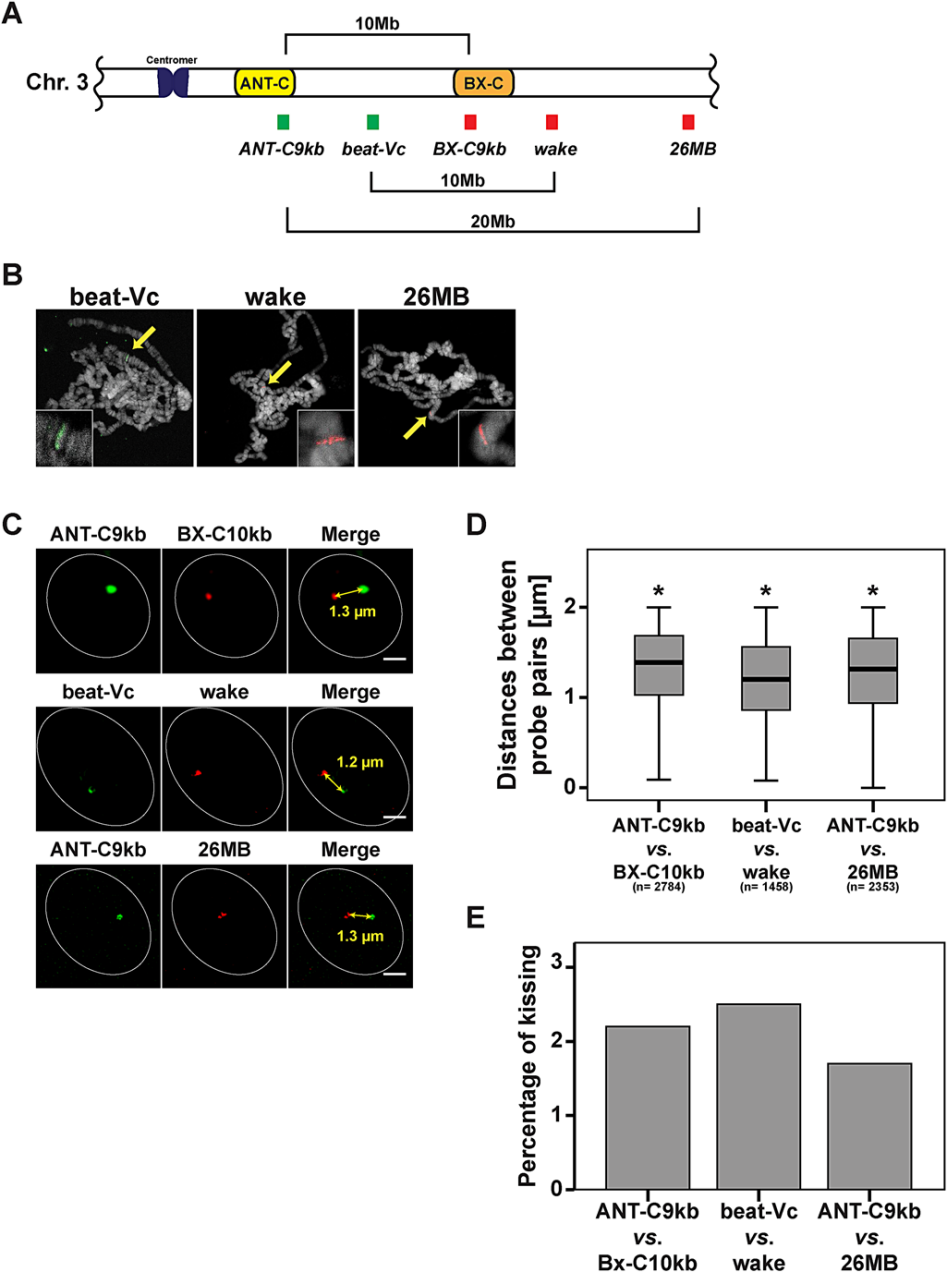
***Hox* gene kissing is a rare event in interphase nuclei of *Drosophila melanogaster.*** (A) Schematic illustration of the location of the positive and negative control probes on chromosome 3R. The colour of the probe indicates the colour used for FISH detection. Beat-Vc and wake have been described as non-kissing sites (Bantignies et al., 2011), and are separated by approximately 10 Mb. The distance of the probe pair ANT-C9kb and 26 MB is twice as long (20 Mb). (B) Specificity of probes verified by DNA FISH on polytene chromosomes. Each of the probes showed a single band (yellow arrow in overview and band in magnified inset). (C) SIM examples of nuclei representing the mean distance between the probes pairs. Mean distances are indicated in the merge view and determined in (D). The scale bars represent 1 µm. (D) Small, but significant differences in the distances between the analysed probe pairs. All three pairs showed an average distance of 1.2 to 1.3 µm. *P<0.05 (two-tailed Mann–Whitney-U test), *n*=total number of analysed nuclei in three different wing imaginal discs. (E) No significant differences in percentage of *Hox* gene kissing for the positive probe pair ANT-C9kb/BX-C10kb and the negative control pairs beat-Vc/wake and ANT-C9kb/26MB. In all cases a range of 1.5% to 2.5% was found.

**Fig. 4.**
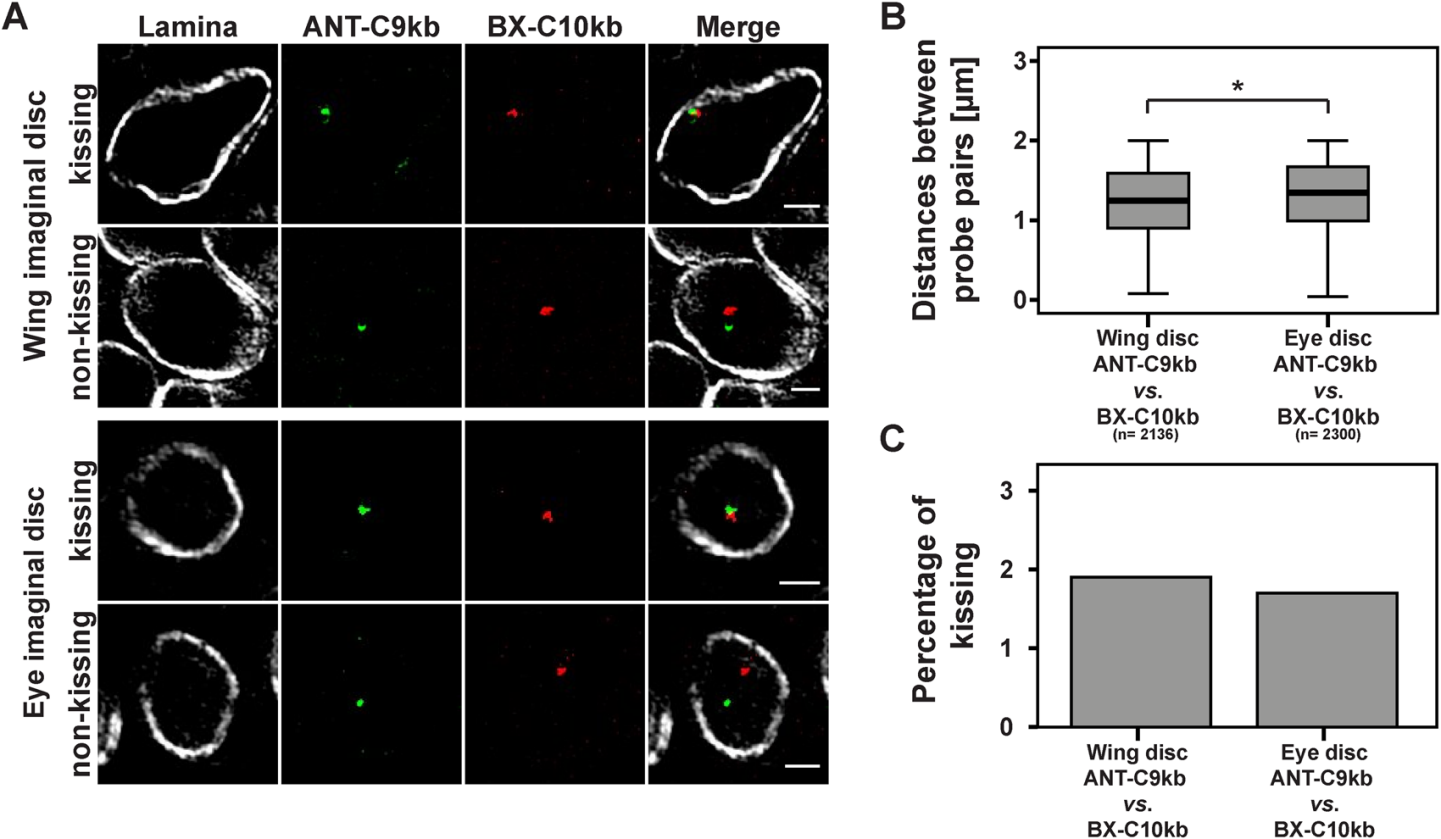
**No differences in the frequency of *Hox* gene kissing events in nuclei of wing and eye imaginal discs.** (A) SIM examples of wing and eye imaginal disc nuclei after hybridization with the ANT-C9kb/BX-C10kb probe pair and combined immune-staining with an antibody against Lamin (Lamina). Nuclei are grouped into kissing and non-kissing cases of the *Hox* genes. The scale bars represent 1 µm. (B) Small, but significant, difference in the mean distance between ANT-C9kb and BX-C10kb in wing imaginal discs and eye imaginal discs. *P<0.05 (two-tailed Mann–Whitney-U test), *n*=total number of analysed nuclei in three different imaginal discs. (C) The percentage of *Hox* gene kissing is in the range of 1.7% to 1.9% and not significantly different in the two tissues.

In order to study insulator speckle formation in the vicinity of contacting (kissing) *Hox* genes, we determined the frequency of kissing events. We used the definition of Bantignies et al. (2011) that the centre of FISH signals should be separated by ≤350 nm in order to indicate a kissing event. With our refined analysis using short probes and high-resolution SIM we expected that the frequency of contacting ANT-C and BX-C sites would be reduced as compared to the published 10% in wing imaginal discs (Bantignies et al., 2011). Indeed the frequency of kissing events in our case was 1.9% (Fig. 2E). Frequencies of the other probe pairs tested above were not significantly different (data not shown). In order to test the proposal that ‘kissing’ probes, as previously detected by confocal laser scan microscopy (CLSM), may in fact turn out to be separated when using the high resolution SIM method, we re-calculated our measurements with a CLSM z-layer resolution. This resulted in a four fold increase in frequencies (Fig. S2), similar to those previously published by Bantignies et al. (2011). Due to the fact that the kissing events are rare, we hypothesized that *Hox* gene kissing might occur at a similar frequency as random interactions. Therefore, we generated control probe pairs, which are proposed not to interact. One such probe pair has been used as a non-interacting negative control. This was the combination of beat-Vc and wake (CG17622) which are located on chromosome 3 and similarly separated by 10 Mb on the linear genome as ANT-C9kb and BX-C10kb (Fig. 3A) and (Bantignies et al., 2011). Another negative control was established by using the probe pair ANT-C9kb and 26 MB. The probe 26 MB is also located on chromosome 3 but separated by approximately 20 Mb (Fig. 3A). FISH on polytene chromosomes revealed high specificity of hybridization at single sites (Fig. 3B). All three combinations, the specific one with ANT-C9kb and BX-C10kb, as well as the negative controls with ANT-C9kb and 26 MB, or with beat-Vc and wake, resulted in significant differences of the mean distance in nuclear space (Fig. 3C,D). Nevertheless, the mean distance reflected the distance between neither the probe sequences in the linear genome nor the fact that ANT-C9kb and BX-C10kb are interacting in 3D. Of these measurements we counted the kissing events and found a range of 1.8 to 2.5%. There was no significant difference between them (Fig. 3E). A similar result was achieved in eye imaginal discs (Fig. S1). Our results indicated that *Hox* gene kissing as determined by the high resolution SIM analysis is as rare as random events observed with the negative controls in interphase nuclei of imaginal wing discs. In contrast to wing imaginal discs, *Hox* gene kissing has been determined to occur at a much higher frequency in imaginal eye discs (Bantignies et al., 2011); therefore, we performed FISH in eye imaginal discs (Fig. 4A). The distances between ANT-C9kb and BX-C10kb are significantly shorter in wing imaginal discs as compared to eye imaginal discs (Fig. 4B). When determining the frequency of kissing in both tissues the percentage was in the range of less than 2% (Fig. 4C). Taken together, our refined FISH analysis with SIM microscopy in wing and eye imaginal discs could not reveal a locus specific increase of contacting (kissing) chromatin regions separated by long distances on the linear genome. Nevertheless, we were convinced by the biochemical evidence of specific *Hox* gene interaction (Bantignies et al., 2011; Cleard et al., 2006; Comet et al., 2006; Lanzuolo et al., 2007; Li et al., 2013; Sexton et al., 2012) and assumed that specific interactions do occur, albeit at a low frequency. Therefore, we decided to further characterize this low percentage of kissing events for a potential association with insulator speckles.

### Insulator speckles and Polycomb bodies are distinct structures

**Fig. 5.**
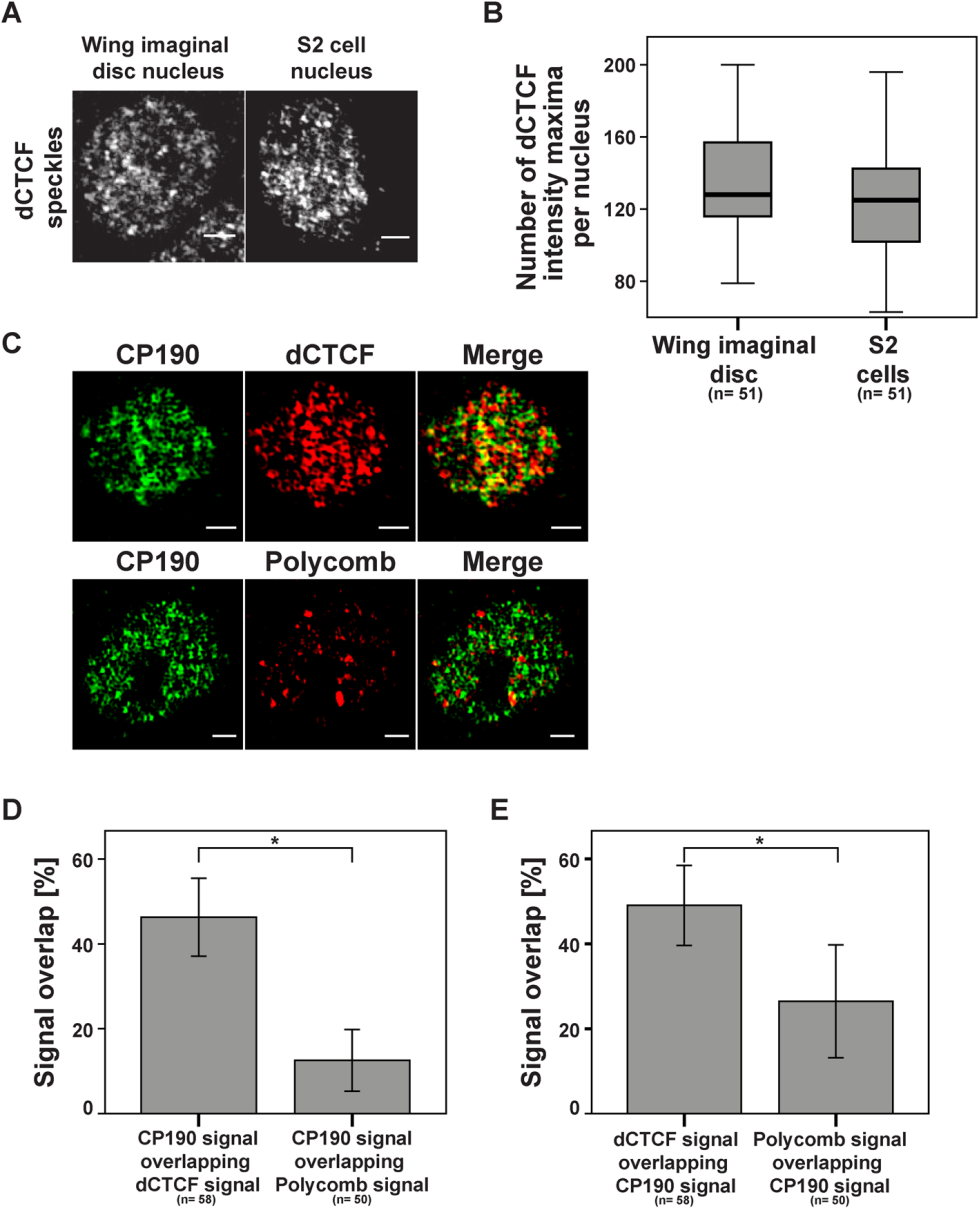
**Insulator speckles are distinct from Polycomb bodies.** (A) Interphase nuclei of *Drosophila melanogaster* wing imaginal disc and of S2 cells after staining with dCTCF antibody and SIM analysis. The speckles are distributed over the whole nucleus and size and density of speckles are similar in the two cell types. The scale bars represent 1 µm. Images illustrate z-projections of the entire nuclei. (B) Both cell types contain nearly the same number of dCTCF insulator speckles (136 speckles vs 128 speckles). (C) S2 cell nuclei are stained with antibodies against CP190 and dCTCF (top row) or against CP190 and Polycomb (bottom row) and SIM analysed. Yellow signals indicate overlapping signals in the merge case. The scale bars represent 1 µm. (D) Significant higher percentage of CP190 signals overlapping with dCTCF as compared to the overlap with Polycomb. (E) The same cells as in (D), but comparison was made between dCTCF signals overlapping with CP190 and Polycomb signals overlapping with CP190. A significantly higher incidence for the dCTCF/CP190 pair was observed. In D and E, *n*=total number of analysed S2 cell nuclei; error bars indicate mean±s.d.; *P≤0.05 two-tailed Mann–Whitney-U test.

Again we used the SIM technique to re-analyse the features of insulator speckles. We did immunofluorescent staining of interphase cells in imaginal wing discs and S2 cells. The number of speckles was determined by counting the number of intensity maxima within the nuclei. About 130 to 140 dCTCF positive speckles were found (Fig. 5A,B). Detection of this high number of speckles was dependent on the resolution of the microscope. When using CLSM, we identified 10 to 20 intensity maxima, whereas the SIM technique resulted in detection of more than 100 speckles (Fig. S3). In order to determine whether the speckle number and morphology may be different in tissue as compared to cell culture we also analysed S2 cells, again about 130 dCTCF positive speckles were counted with a similar appearance as in wing discs (Fig. 5A,B). To further characterize the insulator speckles we wanted to know whether they co-localize with the insulator factor CP190 and whether they are different from nuclear structures called Polycomb group bodies. A potential connection has been seen by demonstrating that insulators are required for long-range interactions between Polycomb targets to form Polycomb bodies (Li et al., 2011); therefore, it might be envisaged that insulator speckles and Polycomb bodies generate joined structures. First, we analysed the overlap between CP190 speckles and dCTCF speckles as well as CP190 speckles and Polycomb bodies (Fig. 5C-E). As expected for the interaction partners dCTCF and CP190 (Bartkuhn et al., 2009; Mohan et al., 2007), nearly 50% of all CP190 speckles co-localized with dCTCF. In contrast to dCTCF, the CP190 overlap with nuclear Polycomb bodies is significantly reduced (Fig. 5D). This reduction can also be seen when comparing the merged (yellow) signals for the CP190/dCTCF pair with CP190/Polycomb (Fig. 5C). To rule out that the significant reduction in colocalization is simply caused by the smaller number of Polycomb bodies as compared to insulator speckles, we determined the percentage of dCTCF speckles overlapping with CP190 and of Polycomb bodies overlapping with CP190 (Fig. 5E). Although there are fewer Polycomb bodies within the nucleus, only 26% of all Polycomb signals overlap with CP190 as compared to 46% of dCTCF speckles, which overlap with CP190. Therefore we can conclude that Polycomb bodies indeed overlap in a fraction of cases (26%), but that in general insulator speckles are not identical to Polycomb bodies.

### Insulator speckles are adjacent to kissing *Hox* genes

**Fig. 6.**
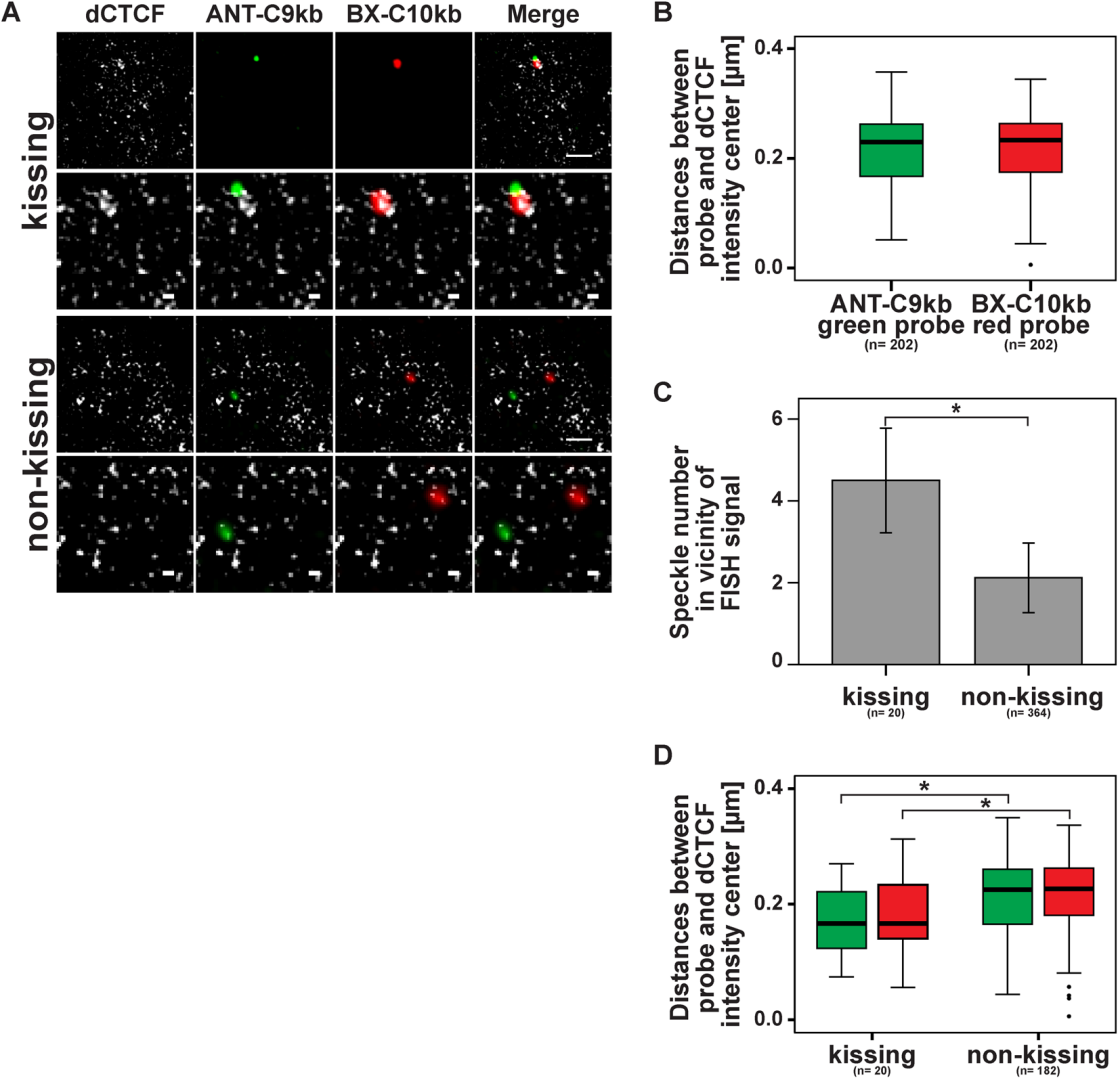
**dCTCF speckles are more close to kissing probes and increased in number.** Wing imaginal disc nuclei were hybridized with the ANT-C9kb/BX-C10kb probe pair and immune-stained with the antibody against dCTCF. After SIM analysis nuclei were grouped into Hox gene kissing cases (probe distance ≤350 nm) and non-kissing cases (probe distance >350 nm). (A) Nuclear examples for kissing (upper image set) and non-kissing (lower image set). The scale bars in the top images of each event represent 1 µm whereas the scale bars in the lower, magnified, images represent 0.2 µm. (B) In all cases, kissing and non-kissing, the distance between the respective FISH probe centre and the closest dCTCF speckle was determined. Without a significant difference, the mean distance of the dCTCF speckles from the ANT-C9kb probe was 0.21 µm and from the BX-C10kb probe was 0.22 µm. (C) The number of dCTCF speckles is increased in case of *Hox* gene kissing events compared to non-kissing events. Error bars indicate mean±s.d.; *P≤0.05 two-tailed Mann–Whitney-U test. (D) As in B, the distance of the dCTCF speckle centre closest to each of the probes was determined, but grouped for kissing and for non-kissing cases. dCTCF speckles are significant closer to kissing probes (*P <0.05; two-tailed Mann–Whitney-U test). *n*=total number of analysed cases.

Having determined that insulator speckles and Polycomb bodies are distinct, we wanted to know whether insulator speckles are sites of insulator interaction or whether they are protein aggregates independent of DNA interaction. Therefore we asked whether insulator speckles could be found at genomic insulator sites and whether this location differs between kissing and non-kissing cases. As distance measurements rely on the quality of the SIM data reconstruction, we applied the SIMcheck procedure (Fig. S4) and found the data being adequately remodelled. Next, we determined the distance between either ANT-C or BX-C FISH probe and the closest dCTCF speckle (Fig. 6A,B). In this analysis we did not distinguish between kissing and non-kissing events. The intensity centre of the FISH probe had a mean distance to the closest dCTCF speckle of 0.21 µm in the case of the ANT-C9kb probe, and a mean distance to the closest dCTCF speckle of 0.22 µm in the case of the BX-C10kb probe (Fig. 6B). This difference was not significant.

Next we determined the number of dCTCF speckles surrounding each of the FISH probes within a distance of ≤350 nm. This local area we call ‘vicinity’. We counted the total number of dCTCF speckles in the vicinity of probe ANT-C or in the vicinity of probe BX-C in the case of non-kissing. In this way we determined the number of speckles in the vicinity of a given spot within the nuclear sphere. This number was 2.1 (Fig. 6C). We compared this to a Monte-Carlo simulation (see Materials and Methods), given that about 130 insulator speckles are found within a nucleus with a radius of 1.5 µm. This simulation predicts that 1.4 speckles will be expected within 350 nm of any given spot in the nucleus. This is similar to the speckle number counted at non-kissing sites, thus determining that the number of insulator speckles at any location within the nuclear sphere is about 2. Then we counted the number of speckles within 350 nm of FISH probes in case of *Hox* gene kissing. Here, we counted 4.5 speckles (Fig. 6C). From this number we can conclude that although the frequency of kissing is low, about 4 insulator speckles mark these sites in distinction from non-kissing cases. Next we wanted to know whether possibly the distance of the speckle closest to the probe used may be different in the case of kissing. This we determined for both of the ANT-C and BX-C FISH probes and found that the distance of the closest speckle was significantly shorter in the kissing cases (0.17 µm and 0.18 µm for the ANT-C and BX-C FISH probes) relative to the non-kissing cases (0.21 µm and 0.22 µm for the ANT-C and BX-C FISH probes) (Fig. 6D).

Taken together, the rare cases of *Hox* gene kissing are singled out by the number and the location of dCTCF insulator speckles within an average of 180 nm in contrast to non-kissing events of *Hox* genes.

## DISCUSSION

For both classes of nuclear factors, Polycomb group proteins (PcGs) and insulator binding proteins (IBPs), a nuclear clustering into bodies or speckles has been observed. For both PcGs and IBPs a functional role in targeting Polycomb-repressed genes into Polycomb bodies has been seen (Bantignies et al., 2011; Li et al., 2013). Here we used the PcG-repressed *Hox* gene clusters ANT-C and BX-C to demonstrate that PcGs and IBPs target to separated nuclear entities, Polycomb bodies and insulator speckles.

In the mouse, *Hox* gene kissing has been observed between individual pairs of genes, although never involving all *Hox* gene clusters simultaneously (Lanctot et al., 2007). In *Drosophila*, there is convincing evidence that the co-repressed ANT-C and BX-C gene clusters interact (Bantignies et al., 2011; Cleard et al., 2006; Comet et al., 2006; Lanzuolo et al., 2007; Li et al., 2013; Sexton et al., 2012). Interaction has been demonstrated by biochemical methods as well as by FISH techniques. The advantage of biochemical methods can be seen in that contact probabilities of specific interactions can be compared to flanking genomic regions and non-specific interactions; however, these methods are based on cell populations and therefore do not reveal the frequencies of interactions within single cells. The latter can be studied with FISH technologies, thus allowing determining the fraction of cells positive for the interaction analysed. The resolution of the FISH technique relies on the microscopic technique used, such that a higher resolution may reduce the frequency of contacting (kissing) genomic regions. Here we used the SIM technique and found that *Hox* gene kissing is quite rare within 1-2% of the cells. This contact frequency is as low as observed for negative control sites. Nevertheless, these rare cases of *Hox* gene kissing must include cases of specific interaction as determined by biochemical methods (Bantignies et al., 2011; Cleard et al., 2006; Comet et al., 2006; Lanzuolo et al., 2007; Li et al., 2013; Sexton et al., 2012). As discussed below, we are providing additional support for specificity by showing that insulator speckles at kissing sites differ from speckles at non-kissing cases.

In order to test whether *Hox* gene interaction does occur at nuclear structures enriched for insulator factors, we had to re-characterise the occurrence of insulator speckles. Previous results clearly demonstrated that insulator bodies are aggregated proteins not involved in insulation (Golovnin et al., 2008, 2012) and that these structures form in response to osmotic stress (Schoborg et al., 2013). Using SIM microscopy, our analysis of the delicate and refined appearance of insulator speckles in the absence of osmotic stress identified about 130 dCTCF and CP190 containing speckles in S2 cells as well as in nuclei of wing imaginal discs. Polycomb bodies have been found to be localized to interacting (kissing) BX-C and *Antp* sequences as well as to non-interacting cases (Grimaud et al., 2006). Furthermore, insulator binding proteins rather than PcG complexes have been found to be involved in the long-range higher-order organization of PcG targets in the nucleus (Li et al., 2011). Our results show that Pc bodies and insulator speckles are clearly distinct and separated, with only 26% of the Pc bodies overlapping with CP190 containing insulator speckles. A conclusion might be that the functional differences between PcGs and insulators in mediating long-range interaction (Li et al., 2011) are conveyed to the cytological level with separated Pc and insulator foci.

In addition to the functional differences of PcGs and IBPs and the distinct nuclear distribution, we found that in case of kissing *Hox* genes the number and the distance of IBP-speckles to the co-localized FISH signals is significantly different from non-kissing cases. This is in contrast to Pc bodies, which colocalize to the *Hox* gene PREs independently of *Hox* gene interaction (Li et al., 2011). As the frequency of kissing *Hox* genes is low, the cases with increased insulator speckle number close to contacting *Hox* genes is similarly low. This supports the above result that there is only little overlap between insulator speckles and Polycomb bodies, which are always binding.

There is a long-standing discussion on the structure, function and dynamic establishment of nuclear foci, such as Polycomb bodies or insulator speckles (Cheutin and Cavalli, 2014; Golovnin et al., 2015; Grimaud et al., 2006; Li et al., 2011, 2013; Mao et al., 2011; Mitchell and Fraser, 2008). The interpretation and discussion of the results obtained in this context is complicated by the potential combination of different features (Mao et al., 2011). Here, within many variants, we may focus on two features. First, focal concentrations of PcG or IBP proteins are sites of long-distance interaction of chromatin. Second, long-distance interaction either causes the focal concentration of PcGs or IBPs or the focal protein concentration may mediate long-distance interaction. For Polycomb bodies it has been seen that focal concentration of PcGs is found at interacting as well as at non-interacting PREs of ANT-C and BX-C (Grimaud et al., 2006). In other words interaction is independent of Polycomb body formation. Furthermore, insulators have been identified to drive Polycomb repressed genes into Polycomb bodies (Li et al., 2011, 2013). In contrast, insulator speckles vary in number and distance in cases of long-distance interaction.

Although we cannot distinguish unequivocally between cause and consequence, we could envisage a causal role for insulator speckles in long-range chromatin interaction. CTCF speckles may nucleate the *Hox* gene interaction. Within a range of 180 nm, these speckles may increase the residence time of interacting regions within a small volume. Such an ‘interaction volume’ possibly containing several interacting regions may explain why, in general, the high-C methods show interaction of a particular site with several alternative interaction partners. Furthermore, a causal role for insulator factors in targeting interacting regions into nuclear substructures, in this case Polycomb bodies, has been demonstrated (Li et al., 2013). Thus, we propose similarities to the ‘transcription factories’, which are of similar size and which combine several genomic regions simultaneously (for review see Edelman and Fraser, 2012).

## MATERIALS AND METHODS

### Fly strain and cell culture

*Drosophila* S2 cells were raised and cultured in Schneider's Medium [Invitrogen; supplemented with 10% fetal bovine serum (FBS), 1% penicillin/streptomycin and glutamine]. *Drosophila melanogaster* flies were maintained on standard medium at 24°C. For the analysis, wild-type strain Oregon R was used.

### Immunostaining

S2 cells were cultured on coverslips for 24 h. After washing with PBS, cells were fixed in 3% paraformaldehyde/PBS followed by several washing steps with PBS. Cells were permeabilized by incubating with 0.5% Triton X/PBS for 5 min on ice.

After blocking in PBS-Tr/10% Normal Goat Serum (NGS) for 30 min at room temperature (RT), cells were incubated with primary antibodies diluted in PBS-Tr/10% NGS for 45 min. Rabbit anti-dCTCF (Mohan et al., 2007), rat anti-CP190 (Golovnin et al., 2012) and rabbit anti-Polycomb (Santa Cruz Biotechnology, sc-25762) were used in 1:1000, 1:2000 and 1:500, respectively. S2 cells were washed in PBS and incubated with secondary antibody (Alexa Fluor conjugated secondary antibodies, Thermo Fisher Scientific) diluted in PBS-Tr/10% NGS for 45 min at RT. DNA was counterstained with Hoechst33342 (AppliChem) and cells were mounted in Fluoromount-G (SouthernBiotech).

### Two-colour 3D DNA FISH and DNA FISH-I

Two-colour DNA FISH was performed as described in Bantignies et al. (2003). Larval imaginal discs were quickly dissected in PBS and fixed in 4% paraformaldehyde/PBT (PBS, 0.1% Tween 20) for 20 min. Tissues were treated with 150 µg/ml RNaseA/PBT for at least 2 h at RT, than incubated in PBS-Tr (0.3% Triton-X) for at least 1 h. In 20 min steps, imaginal discs were transferred into a pre-Hybridization Mixture (pHM; 50% formamide; 4× SSC; 100 mM NaH_2_PO_4_, pH 7.0; 0.1% Tween 20). Imaginal disc DNA was denatured in pHM at 80°C for 15 min. followed by removing of mixture and adding of denatured probes. Before adding, FISH probes were diluted in FISH hybridization buffer (10% dextransulfat; 50% deionized formamide; 2× SSC; 0.5 mg/ml Salmon Sperm DNA) and denatured at 95°C for 10 min. Hybridization was performed overnight at 37°C and 800 rpm agitation. After hybridization, larval imaginal discs were washed with post-hybridization wash solutions.

After post-hybridization washing steps, DNA FISH-I was performed. Larval imaginal discs were blocked in PBS-Tr/10% Normal Goat Serum (NGS) for 2 h at RT, and incubated overnight at 4°C with primary antibody. Rabbit anti-dCTCF (1:1000) (Mohan et al., 2007) and mouse anti-Lamin Dm0 (1:200) (Developmental Studies Hybridoma Bank, ADL67.10) were used. Larval imaginal discs were washed in PBS-Tr, blocked in PBS-Tr/10% NGS for 1 h at RT, and incubated with secondary antibody (Alexa Fluor 647 conjugated secondary antibodies, Thermo Fisher Scientific) for 1 h at RT in PBS-Tr/10% NGS. Larval imaginal discs were mounted in Fluoromount-G (SouthernBiotech). There was no obvious change in number or structure of speckles, when comparing immunostaining with or without FISH procedures.

### Probe description and labelling

FISH probes were purified from whole BAC DNA, PCR fragments amplified from BAC DNA or plasmid fragments. For genomic coordinates see Table S1. Labelled BAC probes covered 150 kb (BacR28H01, BACPAC Resource Centre, Oakland, California, USA) and 190 kb (BacR32J03, BACPAC Resource Centre, Oakland, California, USA) of the Bithorax complex and the Antennapedia complex, respectively. PCR fragments and plasmid fragments covered between 8 kb and 14 kb of the BX-C and the ANT-C, respectively. For testing the specificity of each probe, FISH on polytene chromosomes was carried out (Lavrov et al., 2004).

FISH probes were directly labelled by nick translation using the FISH Tag DNA Green Kit (Alexa Fluor 488 dye, Thermo Fisher Scientific) and the FISH Tag DNA Red Kit (Alexa Fluor 594 dye, Thermo Fisher Scientific). For hybridization on polytene chromosomes, 150 ng of each probe diluted in 15 µl hybridization buffer were used. For hybridization on imaginal discs, 300 ng of each probe diluted in 40 µl FHB were used.

### 3D-SIM microscopy

Imaging was performed using a Zeiss Elyra PS1 system. 3D-SIM data was acquired using a 63×1.4NA oil objective. 488, 561, 642 100 mW diode lasers were used to excite the fluorophores together with respectively a BP 495-575+LP 750, BP 570-650+LP 75 or LP 655 excitation filter. For 3D-SIM imaging a grating was present in the light path. The grating was modulated in 5 phases and 5 rotations, and multiple z-slices were recorded with an interval of 110 nm on an Andor iXon DU 885, 1002×1004 EMCCD camera. Raw images were reconstructed using the Zeiss Zen software. Examples are shown in Fig. S5.

### Data analysis

Measurement of distance between FISH probes was performed with ImageJ (Schneider et al., 2012) in the FIJI framework (Schindelin et al., 2012). A maximum projection was used to identify the lateral position of the FISH probes using the find local maxima algorithm. Subsequently the lateral (z) coordinate was determined in the original 3D stacked image. 3D distance between each ANT-C probe and all BX-C probes was calculated and the nearest neighbour was determined (Tables S3-S8).

For measurement of distance between FISH probes and dCTCF speckles, each probe was marked as a region of interest (ROI) and within this ROI dCTCF maxima were detected. Then distances between dCTCF intensity maxima and the probes were calculated (Table S9). dCTCF positive speckles were determined by counting the number of intensity maxima within a nucleus with ImageJ. To determine the local maxima in the images, a noise tolerance between 40 and 50 was applied. CP190/dCTCF and CP190/Polycomb colocalization analysis were performed with the ImageJ plug-in *JACoP* (Bolte and Cordelieres, 2006) using the standard method Manders' coefficient.

### Statistics

Statistical analyses were carried out using SPSS^®^ (IBM^®^ SPSS^®^ Statistics 22). Differences in distances were analysed by two-tailed Mann–Whitney *U*-test. Significance level is defined as two-tailed asymptotic significance *P*<0.05. Percentage of *Hox* gene kissing was examined by Chi-Square test. Significance level is defined as two-tailed asymptotic significance of chi-square statistic *P*<0.05. Overlap between CP190/dCTCF signal and CP190/Polycomb signal were analysed by one-way analysis of variance, respectively. Significance level is reported as Welch's *F* ratio (*P*<0.05).

For all calculations in the context of long-distance interaction, distances above 2 µm were excluded.

### Monte-Carlo-Simulation

The expected number of speckles at a distance less than 350 nm from a specific position in the nucleus (where either one or two genes are located) in a scenario where both are unrelated and randomly positioned, computer simulations were performed: a sphere with a radius of 1.5 µm (representing the average nucleus in our images) was filled with 130 points (representing the centres of mass of CTCF speckles) randomly picked, using a uniform distribution inside the sphere. Subsequently the number of ‘speckles’ was determined at a distance closer than 350 nm to a position (representing a location where either one or two genes can reside) randomly picked from the same uniform distribution. The simulations were repeated 500 times for 130 speckles, and the average was calculated. The average was 1.4 speckles at a distance smaller than 350 nm for a random position.
